# Inhibiting sirtuin-dependent DNA repair and oxidative stress responses impairs DIPG cell survival

**DOI:** 10.21203/rs.3.rs-7723497/v1

**Published:** 2025-11-21

**Authors:** Sarah A. King, Rana Rheem, Kathryn M. Spitler, Brianne R. O’Leary, Claire H. Graham, Shane R. Solst, Lynn M. Teesch, Sei Sho, James D. Byrne, Douglas R. Spitz, Michelle E. Howard

**Affiliations:** 1. Free Radical and Radiation Biology Program, Department of Radiation Oncology, Holden Comprehensive Cancer Center, University of Iowa, Iowa City, IA, USA.; 2. High Resolution Mass Spectrometry Facility, University of Iowa, Iowa City, IA, USA; 3. Department of Biomedical Engineering, University of Iowa, Iowa City, IA, 52242, USA; Department of Radiation Oncology, University of Iowa, Iowa City, IA, 52242, USA; Holden Comprehensive Cancer Center, University of Iowa, Iowa City, IA, 52242, USA.

**Keywords:** Pediatric Brain Cancer, Oxidative Stress, DNA Damage, DIPG, H3K27M, Histone Deacetylase Inhibitor, Sirtinol, Glioma

## Abstract

**Background:**

Diffuse intrinsic pontine gliomas (DIPG) are universally fatal pediatric brain tumors often characterized by a H3 histone mutation (H3K27M) that leads to epigenetic alterations and gene dysregulation. Histone deacetylases (HDAC), key players in modulating epigenetic pathways in cancer, have emerged as potential therapeutic targets for DIPG. This study evaluates the class III HDAC inhibitor, sirtinol, as a potential therapeutic for DIPG.

**Methods:**

Sirtinol’s efficacy and cellular mechanism were investigated using two patient-derived DIPG cell lines and one immortalized normal human astrocyte (NHAi) cell line as a comparator. Clonogenic survival was assessed under different experimental conditions, and changes in protein expression were measured via western blot and enzymatic assays. Liquid chromatography tandem mass spectrometry (LC-MS-MS) was used to measure brain and blood penetrance of sirtinol in mice.

**Results:**

We found that sirtinol dose-dependently decreased clonogenic cell survival in DIPG cells while having negligible effects on NHAi cells. Combined treatment of sirtinol and radiation resulted in additive toxicity in DIPG, but not NHAi cells. Sirtinol was less toxic in DIPG cells overexpressing mitochondrial-targeted catalase, suggesting peroxide generation as one mode of cell killing. Moreover, sirtinol was found to increase DNA double strand breaks in DIPG cells and decrease phosphorylation of SIRT2-mediated DNA repair pathway kinase ataxia-telangiectasia and Rad3-related protein (ATR). Sirtinol was detected in the brains of sirtinol-treated mice, suggesting blood brain barrier penetrance.

**Conclusions:**

Based on our results, sirtinol shows promise as a selective, redox-modulating therapeutic for DIPG that enhances oxidative stress and interferes with DNA repair.

## Graphical Abstract

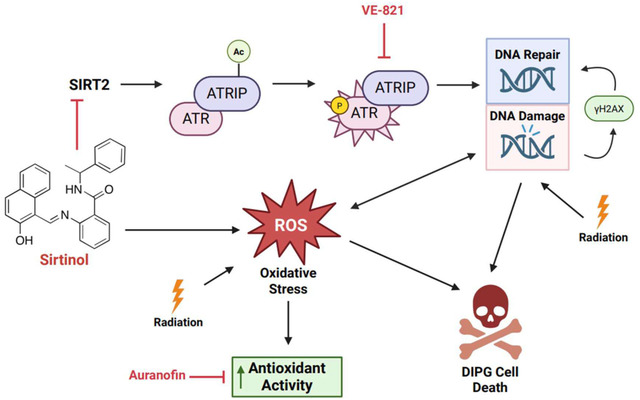

## Background

Diffuse intrinsic pontine glioma (DIPG) is a rare and universally fatal pediatric brain cancer with no sufficient treatment options. The current prognosis is abysmal, with 90% of children diagnosed surviving less than a year post-treatment [1–4]. Although DIPG is rare, with 200–300 annual diagnoses in the United States, it is the leading cause of death among children with brain tumors [4]. Radiation therapy (RT) is the first line of treatment for patients, initially alleviating symptoms. However, these tumors are highly radioresistant, with adaptive changes after RT resulting in eventual relapse and progression following treatment [5]. Additionally, no form of adjuvant chemotherapy has been proven to be superior to RT alone in randomized clinical trials. The universal failure of current therapies to treat patients suffering from DIPG highlights the urgent need for the discovery of novel therapies.

Mutations encoding the histone variants H3.3 (*H3F3A*) and H3.1 (*HIST1H3B* and *HIST1H3C*) have been identified in approximately 80% of patients with DIPG, resulting in a lysine 27-to-methionine (K27M) substitution that affects approximately 80% of patients with DIPG [6–9]. H3K27M prompts a global reduction in trimethylated K27 (H3K27me3), leading to generalized hypomethylation and increased proliferation of tumor cells [10,11]. Given the enhanced oncogenesis due to alterations in epigenetic regulation, cellular signaling changes downstream of H3K27M serve as key therapeutic targets for DIPG. Epigenetic targeting of DIPG using histone deacetylase (HDAC) inhibitors is an emerging treatment strategy supported by an increasing body of preclinical evidence [12–15]. HDACs are enzymes that regulate gene expression by catalyzing the deacetylation of lysine residues on histone tails. Consequently, inhibition of HDACs may have distinct transcriptional effects, such as the restoration of H3K27me3. One concern regarding pan-HDAC inhibitors is their lack of selectivity, showing effectiveness against a range of cancer types, but also having broad effects on normal tissue [16,17]. Initial phase 1 clinical trials using the global HDAC inhibitor Panobinostat (NCT02717455), combined with RT in DIPG showed no improvement in progression free survival or overall survival, in part due to its limited patient tolerability [18]. Considering these findings, identifying HDAC inhibitors with more specific molecular targets may improve outcomes for DIPG patients.

In this context, the sirtuin (SIRT) family of HDACs has risen as a promising target for HDAC inhibition. The sirtuin family comprises seven nicotine adenine dinucleotide (+) (NAD+)-dependent histone deacetylases that are involved in various biological pathways, including cell survival and senescence [19]. Two sirtuins of interest for targeted cancer therapy include SIRT1 and SIRT2, which deacetylate histones H3, H4, and nonhistone substrates, playing a key role in DNA repair and oxidative stress mitigation [20,21]. Regulation of H3 deacetylation by SIRT1 and SIRT2 is particularly relevant for DIPG due to the aberrant gene expression induced by DIPG’s prevalent H3K27M mutation. Depending on the mutational status and tumor type, SIRT1 and SIRT2 can influence both tumor promotion and suppression [22,23]. SIRT2 has been linked to glioma cell proliferation and resistance to DNA damage and oxidative stress by sustaining the cellular redox balance [24]. Further, SIRT2 directly impacts the cellular DNA damage response by regulating the ataxia telangiectasia-mutated and Rad3-related protein (ATR) checkpoint pathway in both cancer and normal cells [25]. SIRT1 is important in the activation of antioxidative and anti-inflammatory pathways, such as those mediated by forkhead box protein O1 (FOXO1) and nuclear factor erythroid 2-related factor 2 (Nrf2) [26–28]. Taken together, there is compelling evidence to suggest that targeted inhibition of SIRT1 and SIRT2 may inhibit DNA repair and reduce the ability of cancer cells to resist oxidative stress induced by radiation.

This study investigates the therapeutic potential of sirtinol, a class III HDAC inhibitor that selectively targets SIRT1 and SIRT2. Sirtinol administration was shown to effectively slow down or temporarily halt tumor growth in a senescence-like growth arrest via the inhibition of SIRT1 function in human lung and breast cancer lines [29, 30]. In addition to halting tumor growth, sirtinol has been found to increase sensitivity to chemotherapy agents in multiple cancer types while having anti-inflammatory effects in normal endothelial tissue, suggesting it may act selectively in cancer vs normal cells [31–35]. In the present study, we explored whether sirtinol will be an effective treatment for DIPG due to its demonstrated antitumor effects in other cancers and its pathologically relevant molecular targets.

## Methods

### Cell lines and culture

Patient derived DIPG cells were obtained from the Monje Laboratory at Stanford University (SU-DIPG50 and SU-DIPG36). The authenticity of the cells was validated with short tandem repeating (Labcorp, Burlington, NC). As described previously [36], a modified tumor stem media (TSM) was used to culture the DIPG cells as monolayers. NHAi cells were obtained from the Koschmann lab at the University of Michigan. NHAi cells were cultured as monolayers in DMEM, 1% N-2, 10% FBS, 1X Antibiotic-Antimycotic, and Normocin (anti-mycoplasma). Media was changed every ~3 d. All cell lines were incubated in 4% O_2_, 5% CO_2_, and held at 37° C. All cells were tested for mycoplasma prior to utilization and every 6 months after.

### Drug treatments

Sirtinol (Selleck, Houston, Tx, S2804) was dissolved in DMSO at 10 mM and used to treat adherent cells for 6 h at doses ranging from 0 to 50 μM. Media was changed at the end of 6 h to end treatment. The ATR inhibitor VE-821 (Sigma, St Louis, MO, SML1415) was dissolved in DMSO at 10 mM and applied to cells for a total of 7 h (added 1 h before sirtinol treatment). Auranofin (Enzo, Farmingdale, NY, BML-EI206) was dissolved in ethanol at 10 mM and used to treat adherent cells at 0.4 μM for 6 h before media change. Doxycycline Hydrochloride (Fisher Scientific, Waltham, MA, BP26535) was dissolved in sterile water at 2 mg/mL. SU-DIPG50-mCat cell flasks were pre-treated with DOX at a concentration of 2 μg/mL for 48 h prior to plating. Adherent cells in 6 well plates were treated at a concentration of 2 μg/mL for 24 h and sirtinol (30 μM, 6 h) was added in the final 6 hours.

For experiments utilizing radiation treatment, sirtinol (30 μM) was applied to pre-plated 6-well plates immediately before irradiation using ^137^Cs (JL Shepherd & Associates, San Fernando, CA). The ^137^Cs source used had a dose rate of 0.5407 Gy min^−1^. Plates were placed at the same distance from the radiation source and time was varied based on the desired Gy. Cells were irradiated at 2, 4, and 6 Gy.

### Clonogenic cell survival

Clonogenic cell survival assays were performed as previously described [36]. Briefly, cells were pre-plated 18 h prior to treatment and incubated with drugs as described above. Cells were incubated for 7–14 days to allow for colony formation (> 50 cells), stained, counted, and analyzed for clonogenic survival compared to control groups.

### Catalase activity assay

The SU-DIPG50-mCat cell line stably expressing a doxycycline-inducible catalase overexpression transgene was developed as previously described [37]. Catalase overexpression was induced in cells with DOX (2 μg/mL) for 48 h prior to drug treatment (24 h DOX ± 6 h, 30 μM sirtinol). Medium was removed, cells were scraped into phosphate buffer (pH 7.0), and pellets frozen at −80 °C. Cells were sonicated and protein concentrations were determined using the Bradford assay. The catalase assay was performed as previously described [60]. The rate of absorption disappearance at 240 nm was measured at 25°C and assays were normalized mg^−1^ cellular protein.

### Western blotting

Following treatment with 30 μM sirtinol, cells (1 × 10^6^) were washed with phosphate buffered saline (PBS) and incubated in lysis buffer with protease inhibitor. Protein from the supernatant was quantified using the BCA protein assay pursuant to manufacturer’s instructions (Thermo Scientific, #23225, St. Louis, MO). 25–40 μg of protein was boiled at 70°C for 10 min, separated by 4–12% gradient SDS-PAGE and transferred onto nitrocellulose or polyvinylidene fluoride membranes. Nonspecific binding was blocked with 5% nonfat dry milk (NFDM) in tris-buffered saline with Tween 20 (TBS-T) for 30 min at RT before incubation with primary antibodies dissolved in 5% NFDM solution at 4°C overnight.

Primary antibodies utilized in this study are as follows: Acetyl Histone H3 (Lys14) (1:500, Cell Signaling Technology, Danvers, MA, 7627), γH2AX (p-Ser139) (1:500, Abcam, Waltham, MA, AB26350), phospho-ATR (Ser428) (1:1000, Cell Signaling Technology, Danvers, MA, 2853), phospho-AKT (1:1000, Cell Signaling Technology, Danvers, MA, 9271)), total-AKT (1:1000, Cell Signaling Technology, Danvers, MA,4685), Nrf2 (1:1000, Cell Signaling Technology, Danvers, MA, 33649) β- Actin (1:2000, Developmental Studies Hybridoma Bank, University of Iowa, JLA20 Actin) and β-tubulin (1:2000, Developmental Studies Hybridoma Bank, University of Iowa, E7).

After primary antibody incubation, membranes were washed with TBS-T and incubated with secondary antibodies dissolved in 5% NFDM solution for 90 min at RT. Secondary antibodies applied include Goat Anti-House IgG HRP (1:10000, AP181P, Sigma-Aldrich, St. Louis, MO) and Goat Anti-Rabbit IgG HRP (1:10000, AP307P, Sigma-Aldrich). Following secondary antibody incubation, membranes were washed with TBS-T before protein detection using enhanced chemiluminescent substrate (34580, Thermo Scientific) and visualization using classic blue autoradiography film. All immunoblots underwent densitometric analysis using ImageJ (version 1.54p) and normalized to a respective loading control. Values are represented as fold change from vehicle treated group.

### Neutral comet assay

Neutral Comet assays were completed using the R&D Systems CometAssay Electrophoresis Starter Kit (#4250–050-ESK, Minneapolis, MN) according to the manufacturer’s instructions, with slight alterations. In short, samples were collected immediately following treatment and resuspended in PBS at 1.5 × 10^5^ cells/mL. Resuspended cells were combined 1:10 with LMagarose and plated onto CometSlides before immersion in pre-chilled lysis buffer (R&D Systems, Inc) overnight at 4°C. The slides underwent electrophoresis in ice cold neutral buffer solution at 1 V/cm for 35 min before sequential incubation in DNA precipitation solution and 70% ethanol. Samples were stained with SYBR Gold (Molecular Probes, Eugene, OR) and visualized using an Olympus BX63 Automated Fluorescence Microscope. After imaging, CometScore (version 2.0.0.38) software was used to determine percent tail DNA and at least 80 comets were quantified in each experimental group.

### Animal Experiments

Female C57BL/6 mice were purchased from Charles River Laboratories (Strain 027, Wilmington, MA, 7–8 weeks). At the end of the treatment period mice were administered ketamine (87.5 mg/kg) and xylazine (12.5 mg/kg) (intraperitoneal, 0.1 ml/20 g) to produce a surgical plane of anesthesia. Once surgical plane was confirmed blood was collected via cardiac puncture and placed in a tube on ice containing EDTA. Confirmation of euthanasia was performed by cervical dislocation in accordance with our animal protocol, pre-approved by the University of Iowa’s Institutional Animal Care and Use Committee (IACUC). Brain samples were collected immediately after euthanasia.

The murine tissue samples collected for mass spectrometry analysis were exclusively from female mice. Since this experiment was designed to evaluate basic pharmacokinetic distribution between plasma and brain samples, sex was not considered a biological variable.

All animal experiments were performed in accordance with the guidelines provided by IACUC. Animals were held in a University of Iowa animal care facility with a 12 h light-dark alternating cycle. Animals were given a 1-week acclimation period prior to the start of treatment and euthanized at the end of the treatment durations before collection of tissue samples.

### Mass spectrometry

After a week of acclimation, mice were administered sirtinol (5 mg/kg) intraperitoneally and then sacrificed 1 or 6 hours after sirtinol treatment. Whole brains were homogenized in DMSO and stored at 80°C, and blood samples were centrifuged for 2000 × g for 10 min before storage of the plasma supernatant at 80°C.

Upon thawing, 200 μL acetonitrile containing the internal standard phenacetin (100 ng/mL) was added to the brain samples and vortexed for 30 seconds before centrifugation at 7000 × g for 10 min at 4°C. The supernatant was diluted 1:2 in a new tube, vortexed and centrifuged at 14000 × g for 10 min at 4°C and moved into an autosampler vial for analysis. Plasma samples were similarly thawed and vortexed with 120 μL of acetonitrile containing phenacetin (100 ng/mL) and placed in the dark on ice for 10 min before quick vortex and centrifugation at 14000 × g for 10 min at 4°C. 50 μL of the plasma supernatant was moved to an autosampler vial for analysis. LC-MS-MS data was collected using a Water Xevo TQ-S Cronos with an Acquity H-Class UPLC system. An Aglient Zorbax Eclipse Plus C8 LC column (2.1 mm × 100 mm, 1.8 μm) was used with a flow rate of 0.2 mL/min and an injection volume of 5 μL. The column was held at 30°C throughout the run. For the mobile phase, solvent A was water with 0.1% formic acid and solvent B was acetonitrile with 0.1% Formic Acid. The LC gradient started at 60% Solvent B and held for 6 minutes then increased to 95% B in 1 minute, where it was held for 2 minutes prior to re-equilibration. Data was collected using positive electrospray. The (M+H)^+^ ion to product ion (selected reaction monitoring or SRM) transitions used were 395.14 >291.17, 230,12, and 105.05 for sirtinol and 180.13 > 138.07 and 110.02 for phenacetin.

### Statistics

Statistical analysis was performed using GraphPad Prism version 10.4.0 for Windows (GraphPad Software, Boston MA), with statistical significance set at p < 0.05 and 95% CI for all analyses. Each experiment consists of at least three separate biological replicates (n ≥ 3). Unpaired t-test was used for analysis of two means, and one-way ANOVA with Fisher’s LSD was used for analysis of three or more means.

## Results

### Sirtinol decreases DIPG clonogenic cell survival and is additive with RT

**Figure 1.**
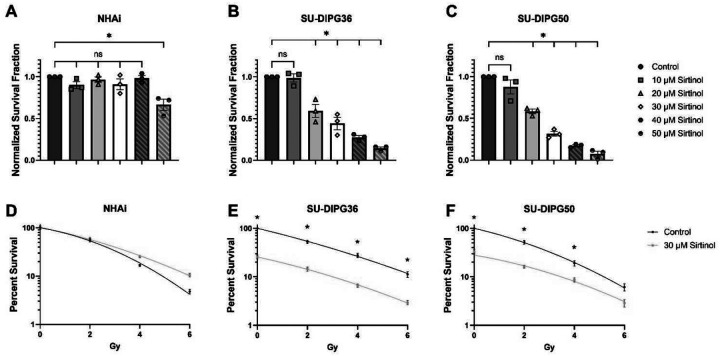
Sirtinol decreases DIPG clonogenic cell survival and is additive with RT. Pre-plated clonogenic survival assays were performed in untreated (control) or sirtinol-treated (10–50 μM, 6h) NHAi (A), SU-DIPG36 (B) and SU-DIPG50 (C) cells. To assess radiation effects, pre-plated clonogenic survival assays were performed in untreated (control) or sirtinol-treated (30 μM, 6h) NHAi (D), SU-DIPG36 (E) and SU-DIPG50 (F) cells and then irradiated (0–6 Gy). Survival is normalized to untreated control. Data are shown as mean ± SEM of three experiments with three replicates (n=3–4, N=9–12). *P < 0.05, ns = not significant.

Pre-plated clonogenic cell survival assays were used to determine sirtinol’s ability to selectively target DIPG vs normal human astrocytes (NHAi). Sirtinol did not significantly affect NHAi survival until the maximum dose tested of 50 μM (Fig. 1A). In contrast, cell survival dose-dependently decreased in both SU-DIPG36 (Fig. 1B) and SU-DIPG50 (Fig. 1C), starting at 10 μM of sirtinol and continuing until the maximum dose of 50 μM. To assess the combined effect of sirtinol and RT, a dose of 30 μM sirtinol and a treatment duration of 6 h was chosen.

Clonogenic survival progressively decreased following RT alone in all cell lines tested (Fig. 1D–F). Treatment with sirtinol + RT did not significantly affect the survival of NHAi cells compared to RT alone, even showing a slight (but not statistically significant) protective effect in these cells at 4 and 6 Gy (Fig. 1D). In contrast, sirtinol-treated SU-DIPG36 cells exhibited a significant additive increase in cell death at every RT dose examined when compared to RT alone (Fig. 1E), and sirtinol-treated SU-DIPG50 cells showed significant additive toxicity at 0, 2, and 4 Gy, but not 6 Gy. (Fig. 1F). These findings confirm that sirtinol is selectively toxic to DIPG cells and additive with radiation treatment.

### Sirtinol inhibition of sirtuins increases acetylation of histone H3 in DIPG cells

**Figure 2.**
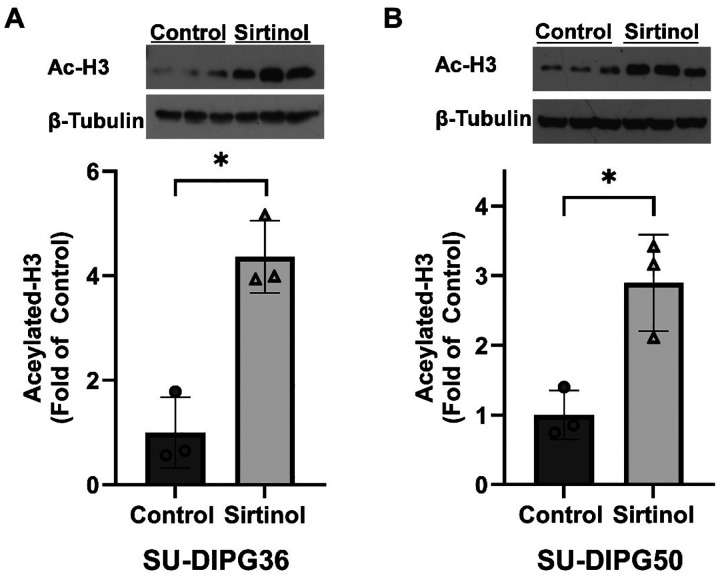
Treatment with sirtinol increased histone H3 acetylation in DIPG cells. Protein expression of Acetylated-H3 in SU-DIPG36 (A) or SU-DIPG50 (B) following treatment with either vehicle (dimethyl sulfoxide (DMSO)) or sirtinol (30 μM, 6 h) (n=3). Data are shown as mean ± SD of three experiments. * P < 0.05, from unpaired *t* tests. Full length blots are presented in Supplemental Figure 2.

Considering sirtinol is reported to be a selective SIRT1 and SIRT2 inhibitor, we assessed the changes in acetylated H3 (Ac-H3) protein expression in sirtinol-treated SU-DIPG50 and SU-DIPG36 cells. Compared to vehicle-treated controls, Ac-H3 significantly increased following treatment with sirtinol (30 μM, 6 h) in both SU-DIPG36 (Fig. 2A) and SU-DIPG50 (Fig. 2B) cells. These data confirm that sirtinol was able to inhibit H3 deacetylation in DIPG cells.

### Sirtinol targets redox homeostasis via peroxide generation and antioxidant interference

**Figure 3.**
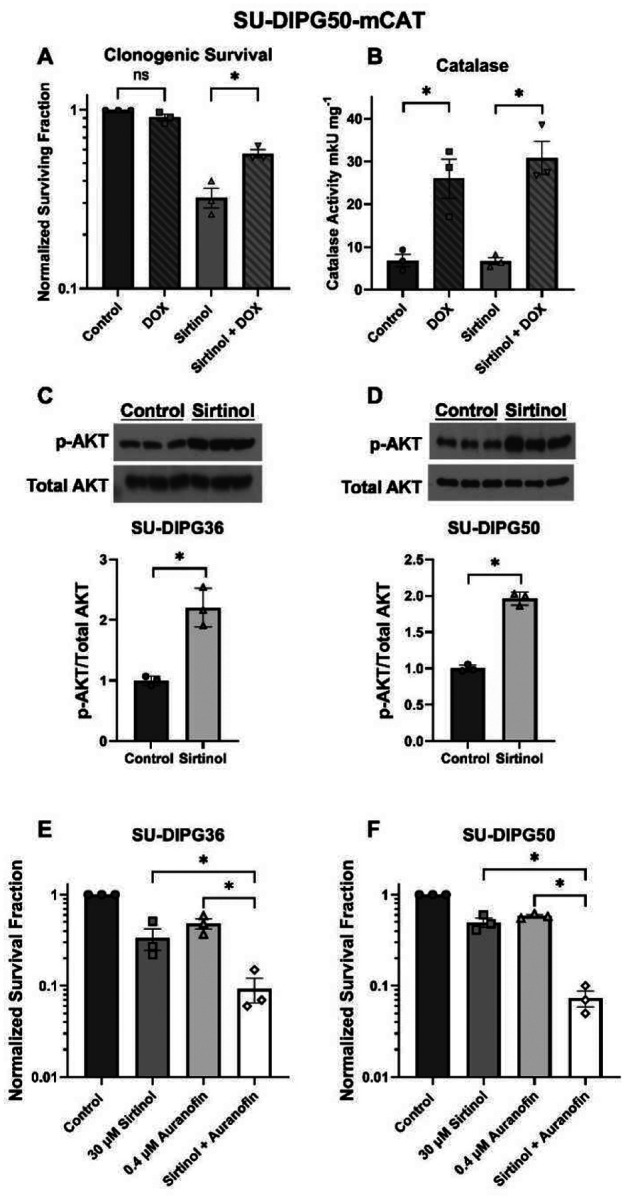
Sirtinol-induced cytotoxicity is mediated by hydrogen peroxide and can be enhanced by inhibiting hydroperoxide metabolism. **(A)** Pre-plated clonogenic survival assays were performed using SU-DIPG50-mCAT cells ± doxycycline (DOX; 2 μg/mL, 48 h pretreatment, 24 h post-plating) and ± sirtinol (30 μM, 6 h). Survival fraction is normalized to untreated control. **(B)** Catalase activity was measured in SU-DIPG50-mCAT cell homogenates ± DOX (2 μg/mL, 48 h) and ± sirtinol (30 μM, 6 h). Catalase activity was normalized to total protein. Data are shown as mean ± SEM of three experiments plated in triplicate (n=3, N=9). **(C-D)** Western blot analysis of p-AKT and total AKT expression in SU-DIPG36 (C) and SU-DIPG50 (D) cells treated with either vehicle control (DMSO) or sirtinol (30 μM, 6 h). **(E-F)** Clonogenic survival of SU-DIPG36 (E) and SU-DIPG50 (F) cells treated with sirtinol, auranofin, or both drugs combined. Data are normalized to untreated control. *p < 0.05. ns = not significant. Full length blots are presented in Supplemental Figure 2.

SU-DIPG50-mCAT cells containing a doxycycline-inducible catalase-overexpressing transgene were used to determine whether hydrogen peroxide (H_2_O_2_) generation is involved in sirtinol’s anticancer effects. Clonogenic survival was assessed in SU-DIPG50-mCAT cells after 6 h sirtinol treatment (30 μM) and ± doxycycline (DOX: 2 μg/mL, 48 h pretreatment + 24 h post-plating treatment). Induction of catalase using DOX significantly decreased sirtinol’s toxicity, confirming that H_2_O_2_ plays a role in sirtinol-induced DIPG cell death (Fig. 3A). Catalase induction was confirmed in cells treated with and without sirtinol ± DOX via a catalase activity assay. Treatment of SU-DIPG50-mCAT cells with DOX resulted in a significant 3-fold increase in catalase activity, while catalase activity was unchanged in cells treated with sirtinol alone (Fig. 3B).

Due to its role in cellular redox regulation and response to oxidative stress, we measured sirtinol’s effect on total AKT and phosphorylated AKT (p-AKT) expression in SU-DIPG36 (Fig. 3C) and SU-DIPG50 (Fig. 3D) cells via western blot. While total AKT levels did not change after sirtinol treatment (30 μM, 6 h), sirtinol significantly increased p-AKT levels in both DIPG cell lines. Levels of Nrf2, a transcription factor that is involved in regulating antioxidant activity, were measured via western blot to further verify that sirtinol may act as a pro-oxidant therapeutic. SU-DIPG50 and SU-DIPG36 cells exhibited significantly higher total Nrf2 levels following treatment with sirtinol (6 h, 30 μM), indicating a cellular antioxidant response to reactive oxygen species (ROS) generation from sirtinol (Suppl. Fig. 1).

Next, we assessed whether the addition of auranofin, an inhibitor of thioredoxin reductase (TrxR) which is a key enzyme in hydroperoxide metabolism [38], could enhance sirtinol-induced cytotoxicity in DIPG cells. Clonogenic survival was measured in SU-DIPG36 (Fig. 3E) and SU-DIPG50 (Fig. 3F) cells treated with sirtinol (30 μM, 6 h) ± auranofin (0.4 μM, 6 h). Treatment of DIPG cells with sirtinol and auranofin together resulted in additive killing, wherein cell survival was significantly lower in combination-treated cells than in cells that received either agent alone.

### Sirtinol increases DNA double-strand breaks

**Figure 4.**
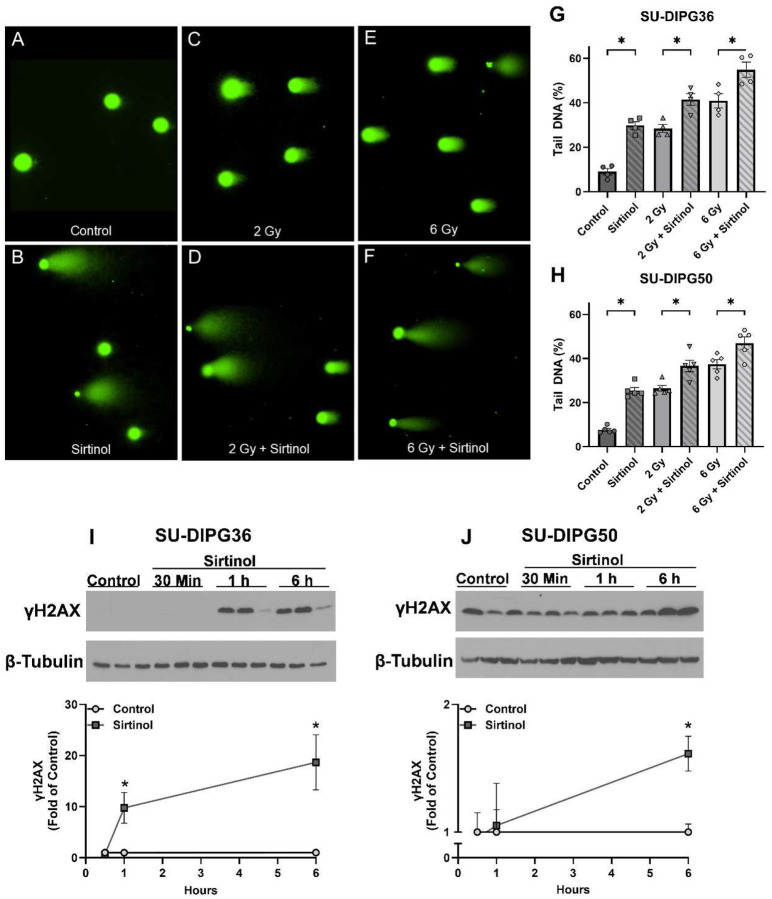
Sirtinol increases double stranded DNA breaks in DIPG cells. (A-F) Representative images of SU-DIPG50 neutral comet assay samples, obtained from the same biological replicate for each treatment group. (G-H) Neutral comet assays were performed in SU-DIPG36 (G) and SU-DIPG50 (H) cells treated ± RT (0, 2, or 6 Gy) and ± sirtinol (30 μM, 6 h before RT). Bars represent mean percent tail DNA ± SEM. n=4–5 for each treatment condition. (I-J) Western blot analysis of γH2AX expression in SU-DIPG36 (I) and SU-DIPG50 (J) cells treated with vehicle control (DMSO) or sirtinol (30 μM, 30 min, 1 h, 6 h) (n=3). Data are expressed as mean ± SD. *P < 0.05. Full length blots are presented in Supplemental Figure 2.

To determine the effect of sirtinol and RT on DNA damage, we measured DNA double-strand breaks in SU-DIPG50 and SU-DIPG36 cells using the neutral comet assay. Cells were treated with 30 μM sirtinol 5.5 h before RT (2, 6 Gy) and harvested immediately following RT. Sirtinol directly increased DNA double strand breaks as indicated by a significant increase (p < 0.05) in percent tail DNA compared to control and/or RT alone (Fig. 4A–H). To identify changes in a DNA-damage response marker in sirtinol-treated DIPG cells, histone H2AX phosphorylation of serine 139 (γH2AX) was assessed after treatment with sirtinol (30 μM) for 30 min, 1 h and 6 h (Fig. 4I, J). γH2AX is a sensitive biomarker for DNA double-strand breaks [39]. There was an increase in γH2AX beginning at 1 h and continuing at 6 h in the SU-DIPG36 cell (Fig. 4I). In the SU-DIPG50 cells, a significant increase in γH2AX was not observed until after 6 h of treatment (Fig. 4J).

### SIRT2 inhibition by sirtinol effects the ATR pathway in DIPG cells

**Figure 5.**
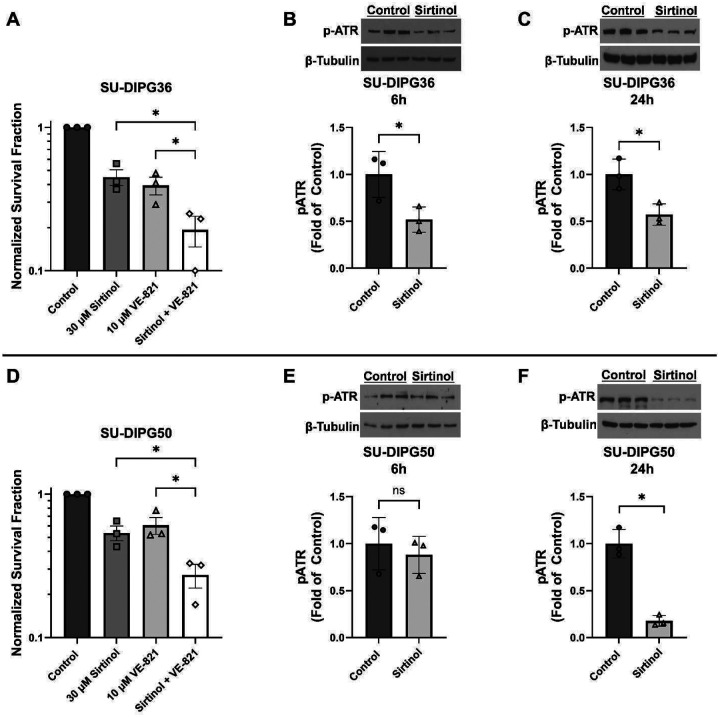
Sirtinol interferes with ATR-mediated DNA repair. (A,D) Clonogenic survival of SU-DIPG36 (A) and SU-DIPG50 (D) cells treated with sirtinol (30 μM, 6 h) or VE-821 (10 μM, 7 h), or both drugs combined. Data are normalized to untreated controls and represented as mean survival fraction ± SEM (n=3). Western blot analysis of phosphorylated ATR (p-ATR) expression in SU-DIPG36 and SU-DIPG50 cells treated with either control (DMSO) or sirtinol (30 μM) for (B,E) 6 h or (C, F) 24 h. Data are expressed as mean ± SD. *P < 0.05. ns= not significant. Full length blots are presented in Supplemental Figure 2.

Given the ability of SIRT2 to regulate ATR, a kinase known for its role in DNA repair [25], the ATR pathway became a target for mechanistic study. Phosphorylated ATR (p-ATR) levels were assessed via western blot in both SU-DIPG36 and SU-DIPG50 cells treated with 6 h or 24 h sirtinol (30 μM) and compared to a vehicle (DMSO) control. Expression of p-ATR was significantly lower in SU-DIPG36 cells treated with sirtinol at both 6 and 24 h compared to vehicle-treated cells (Fig. 5 B–C). In SU-DIPG50 cells, p-ATR expression was not significantly different after 6 h sirtinol treatment (Fig. 5E) but showed a significant decrease after 24 h sirtinol treatment (Fig. 5F). Given the changes in p-ATR levels observed after sirtinol treatment, we examined whether pairing sirtinol with an ATR-inhibiting compound would result in additive cell killing. Clonogenic survival of SU-DIPG36 and SU-DIPG50 cells was measured following treatment with sirtinol, the ATR inhibitor VE-821, or both drugs combined. Surviving fraction was significantly decreased in both DIPG cell lines treated with sirtinol and VE-821 combined compared with toxicity observed from either agent alone (Fig. 5A–B).

### Sirtinol is present in mouse brain and plasma samples following intraperitoneal administration

**Figure 6.**
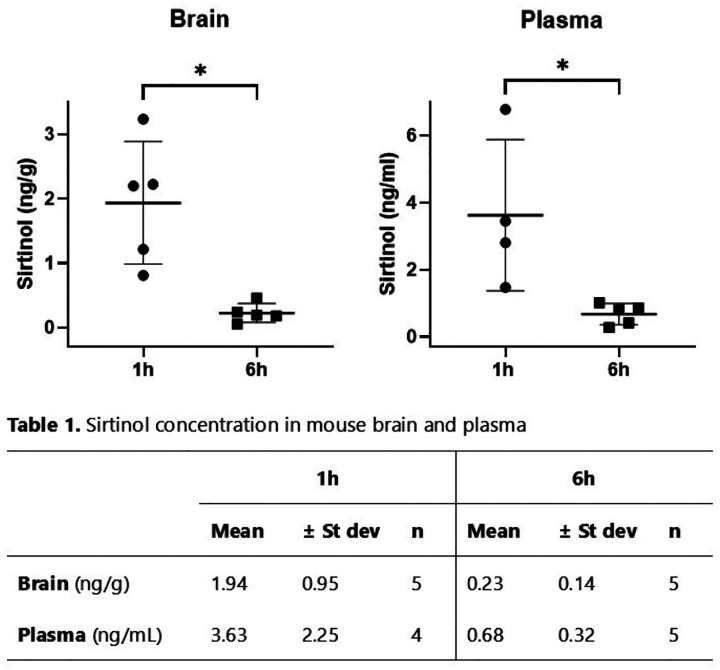
Concentrations of sirtinol in mouse brain and plasma after intraperitoneal administration. Female C57Bl/6J mice were treated with sirtinol (5 mg/kg, i.p.) and sacrificed 1 h (n=5) or 6 h (n=5) after treatment. Brain and plasma samples were harvested, processed and analyzed by liquid chromatography–mass spectroscopy to determine sirtinol concentrations. Graphed data depict individual sirtinol concentrations for each mouse sample, with error bars representing the mean ± SD for each time point. Table 1 summarizes the LC-MS-MS data as the mean ± SD for each tissue type and treatment duration. *P < 0.05.

As a preliminary assessment of sirtinol’s distribution in mouse brain and plasma, tissue samples were collected from mice 1 and 6 h after intraperitoneal administration of sirtinol (5 mg/kg) and analyzed using liquid chromatography tandem mass spectrometry (LC-MS-MS). There was an average sirtinol concentration of 1.94 ng/g in the brain samples collected 1 h after treatment (n=5), and only trace amounts measured in the samples collected 6 h after treatment (n=5). In plasma, the average sirtinol concentration was 3.63 ng/mL in the 1h samples (n=4), and 0.68 ng/mL (n=5) in the 6 h samples (Fig. 6).

## Discussion

Due to DIPG’s unique epigenetic profile and resistance to conventional treatment modalities, targeted HDAC inhibition may be a promising treatment strategy for this disease. Limited tolerability in DIPG patients treated with panobinostat the phase I clinical trials [18] highlights the need for HDAC inhibitors with highly specific targets. This study examines the therapeutic potential of the SIRT1 and SIRT2 inhibitor, sirtinol, in DIPG with or without standard-of-care RT.

In this study, we provide evidence that sirtinol effectively and dose-dependently reduces DIPG cell survival without significantly impacting NHAi cells. The effects of 30 μM sirtinol on DIPG were additive when paired with RT, affirming that sirtinol’s cytotoxic effects combine with standard-of-care radiation to further decrease tumor cell survival. While previous work has shown sirtinol reducing cytokine-induced inflammation in dermal endothelial cells [33], its selectivity in cancer vs normal cells was previously not well-characterized. These results affirm that sirtinol exhibits selective toxicity to the DIPG cell lines tested and not to normal NHAi cells with and without RT, highlighting its potential as a selective therapeutic agent.

Since sirtinol has been shown to specifically inhibit SIRT1 and SIRT2 [40–42], we measured levels of Ac-H3, a downstream target that is in part regulated by SIRT1 [43], involved in tumorigenesis, and characteristically mutant in 80% of DIPG tumors. Sirtinol treatment resulted in significant increases in Ac-H3 in SU-DIPG36 and SU-DIPG50 cells, confirming successful inhibition of histone deacetylation. Increased H3 acetylation has been linked to improved immune activity against DIPG [44]. The specific H3 target probed in this study (H3K14ac) is selectively associated with H3K27me3 [45], suggesting potential restoration of H3K27me3 proportional to sirtinol-mediated ac-H3K14 increases. H3K27me3 is a post-translational modification that is dramatically reduced in H3K27M-mutated DIPG tumors, contributing to epigenetic dysregulation [46]. Increases in H3K14ac are also linked specifically to HDAC inhibition, further supporting sirtinol is a selective HDAC inhibitor.

Dysregulated ROS metabolism is a primary characteristic of virtually all cancers, with moderate accumulations of ROS bolstering survival and proliferation of cancer cells [47–49]. Many cancer therapies seek to upset this altered redox homeostasis by inducing ROS accumulation, in turn leading to cancer cell death [50–52]. Our results suggest that sirtinol treatment may decrease cell survival by upsetting cellular redox balance in DIPG. SU-DIPG50-mCAT cells were protected from sirtinol in the presence of DOX-induced catalase overexpression, indicating H_2_O_2_-induced cell death as one component of sirtinol’s cytotoxicity. Additionally, hydroperoxide metabolism inhibition using auranofin resulted in additive toxicity with sirtinol, suggesting complementary mechanisms involving ROS generation and inhibition of ROS metabolism. This treatment modality is particularly relevant considering radiation, the sole life-prolonging intervention for DIPG, induces oxidative stress via ROS generation [53,54], highlighting sirtinol’s potential to build upon standard-of-care RT in DIPG.

Due to SIRT1’s role in activating various cellular antioxidant pathways [55–57], we expected treatment with sirtinol to decrease levels of SIRT1-mediated antioxidant signaling proteins such as Nrf2 and p-AKT. Instead, sirtinol increased Nrf2 and p-AKT in both DIPG cell lines tested, suggesting a potential cellular response to ROS buildup and increased oxidative stress. Excessive oxidative stress can activate PI3K/AKT signaling [58], promoting increased ROS production and inhibiting FoxO-mediated antioxidant pathways, ultimately leading to cell death [59, 60]. Thus, sirtinol treatment may create an ideal cellular environment for AKT-mediated apoptosis induction via oxidant stimuli. In addition, the increased DNA double-strand breaks recorded after treatment of DIPG cells with sirtinol ± RT provide evidence of ROS-mediated DNA damage. Increases in γH2AX were not immediate- appearing after 1 h of sirtinol treatment in SU-DIPG36 cells and after 6 h in SU-DIPG50 cells, suggesting that the damage is not due to direct genotoxic effects.

We hypothesized that inhibiting SIRT2 with sirtinol would interfere with ATR-mediated DNA repair, contributing to sirtinol’s cytotoxicity. SIRT2 has been shown to deacetylate the ATR regulatory partner ATRIP, which drives autophosphorylation of ATR and facilitates DNA repair in response to replication stress [25]. The decrease in p-ATR observed in both DIPG cell lines provides evidence that targeted inhibition of SIRT2 using sirtinol influences the ATR checkpoint pathway by preventing the activation of ATR. The additive cell killing observed in DIPG cells treated with sirtinol and the ATR inhibitor VE-821 confirm that sirtinol’s toxicity is not solely ATR-specific DNA repair inhibition.

Additional work on sirtinol is needed to optimize its translation into DIPG. While we were able to confirm H_2_O_2_ as one facet of sirtinol’s toxicity, it is unclear whether sirtinol directly generates H_2_O_2_ or indirectly causes a buildup via other means. Further, there may be additional ROS in addition to H_2_O_2_ contributing to sirtinol-induced oxidative stress. Additionally, despite our LC-MS-MS results providing evidence of sirtinol’s ability to cross the blood brain barrier, further research is needed to fully characterize sirtinol’s pharmacokinetics in the brain. Lastly, while the two DIPG cell lines used in this study, SU-DIPG36 and SU-DIPG50, possess the primary histone mutations in DIPG (H3.1 and H3.3, respectively), these cell lines do not fully represent the diverse genotypes present in DIPG tumors. Future studies assessing sirtinol’s therapeutic effects on additional cell lines, as well as its efficacy *in vivo*, are warranted.

### Conclusions

Overall survival of patients with DIPG has not improved despite decades of scientific discoveries, underscoring the critical need to characterize novel treatment options for this disease. In this study, we have shown sirtinol to be selectively toxic to DIPG cells by modulation of intracellular ROS, DNA damage induction, DNA repair inhibition, and/or a combination of these mechanisms. While further research is necessary to determine the clinical applications of sirtinol, the results of this study may serve as a basis for its development as an effective therapeutic agent in DIPG.

## Supplementary Material

1

Supplementary Files

This is a list of supplementary files associated with this preprint. Click to download.

- SupplementalFig.1.tif
- SupplementalFigure2UncroppedWesternsFinal.pdf

## Data Availability

The data supporting the findings in this study are included in this article and its supplementary material. Additional raw data are available upon reasonable request to the corresponding author.

## Abbreviations:

AKT: protein kinase B
ATR: ataxia-telangiectasia and Rad3-related protein
RT: radiation therapy
DIPG: diffuse intrinsic pontine glioma
DMSO: dimethyl sulfoxide
DOX: doxycycline
FOXO1: forkhead box protein O1
HDAC: histone deacetylase
H_2_O_2_: hydrogen peroxide
LC-MS-MS: Liquid chromatography tandem mass spectrometry
NAD+: nicotine adenine dinucleotide (+)
NFDM: nonfat dry milk
NHAi: immortalized normal human astrocytes
Nrf2: nuclear factor erythroid 2-related factor
ROS: reactive oxygen species
SIRT: sirtuins
TrxR: thioredoxin reductase
TSM: tumor stem media
