# Supplementary material for: Inhibiting sirtuin-dependent DNA repair and oxidative stress responses impairs DIPG cell survival
Source: Res Sq. 2025 Nov 21:rs.3.rs-7723497. Preprint. [Version 1] doi: 10.21203/rs.3.rs-7723497/v1 (PMC12668155; doi:10.21203/rs.3.rs-7723497/v1)
Supplement: 1 [file NIHPPRS7723497V1-supplement-1.pdf]

Supplemental Data

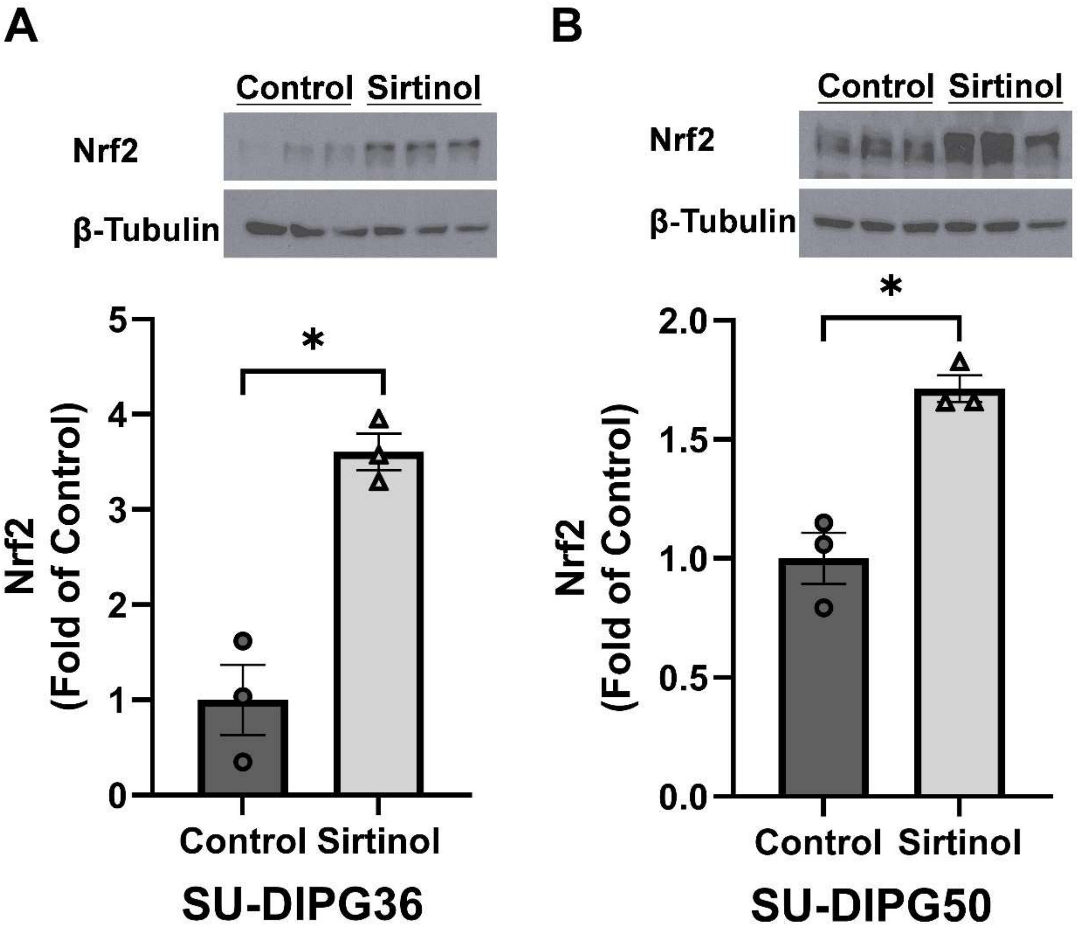

Supplemental Figure 1. Sirtinol increased Nrf2 expression in DIPG cells. Protein expression of Nrf2 in SU-DIPG36 (A) or SU-DIPG50 (B) following treatment with either vehicle (DMSO) or sirtinol (30 μM, 6 h) (n=3). Data are shown as mean ± SD of three experiments. * $P < 0.05$, from unpaired *t* tests.
